# 127 Barriers, Facilitators and Opportunities to Promote Walking in Middle and Older-Aged Adults

**DOI:** 10.1093/eurpub/ckae114.043

**Published:** 2024-09-26

**Authors:** Alison O’Meara, Heather Kilbride, James Grace, Laura Maye, Paul Holloway, Rosane Mingham, Jason Chan, Anna O’Reilly Trace, Annalisa Setti

**Affiliations:** School of Applied Psychology, University College Cork, Cork, Ireland; School of Applied Psychology, University College Cork, Cork, Ireland; Department of Geography, University College Cork, Cork, Ireland; School of Computer Science and Information Technology, University College Cork, Cork, Ireland; School of Computer Science and Information Technology, University College Cork, Cork, Ireland; Department of Geography, University College Cork, Cork, Ireland; School of Computer Science and Information Technology, University College Cork, Cork, Ireland; School of Applied Psychology, University College Cork, Cork, Ireland; School of Applied Psychology, University College Cork, Cork, Ireland; School of Applied Psychology, University College Cork, Cork, Ireland

## Abstract

**Purpose:**

Physical activity is a key factor in preventing dementia (Livingston et al., 2020) and reducing the risk of other non-communicable diseases (Ding et al., 2016). Almost 50% of middle-aged Irish people do not meet the recommended guidelines for physical activity; people still use functional walking limitedly and the car is a preferred means of transport (Healthy Ireland, 2019; National Household Travel Survey, 2022). The aim of this study was to capture perceptions of the barriers, facilitators and potential solutions to promote walking across different stakeholders, aiming to increase walking in middle-older age.

**Methods:**

Perceptions were gathered across five distinct stakeholder groups (Walkers, Academics, Healthcare Professionals, Policy Makers, Charity/Community Groups) via recorded, semi-structured interviews (n = 42) and focus groups (n = 3).

**Results:**

Results were analysed using thematic analysis. Stakeholders indicated that any intervention aimed to promote walking needs to be tailored to specific groups; any tool used (technology-based or not) needs to account for a spectrum of needs and motivations. Recreational and functional walking are perceived differently; functional walking, coinciding with city walking, is perceived as a loss of time by middle-aged people, older adults are more positive, although barriers are present. Recreational walking is perceived as health enhancing by all stakeholders, but with negative environmental impact by policy makers and advocates aiming at reducing carbon emissions. All stakeholders are open to technological solutions to address the issue of increasing health-enhancing and community-beneficial walking, but interactive solutions are needed.

**Conclusions:**

This study provides valuable insights into the key barriers and facilitators to walking amongst middle-aged and older adults, which can help to inform tailored solutions that aim to promote walking. The findings also bear relevance to policy makers aiming to promote more sustainable communities, as they shed light on when, where and for whom walking is beneficial, and how people feel about the type of environment they walk in.

**Support/Funding Source:**

This project is funded through the Science Foundation Ireland National Challenge Fund.

